# Suicidal ideation and HIV risk behaviors among a cohort of injecting drug users in New Delhi, India

**DOI:** 10.1186/1747-597X-8-2

**Published:** 2013-01-15

**Authors:** Enisha Sarin, Basant Singh, Luke Samson, Michael Sweat

**Affiliations:** 1 Independent consultant, B7, 1st floor, Suncity, Sector 54, Gurgaon, 122002, Haryana, India; 2 Department of Psychiatry and Behavioral Sciences, Family Service Research Center, The Medical University of South Carolina, McClennan Banks 4th Floor, 326 Calhoun St. STE MC406, Charleston, SC, 29401, USA; 3 Sharan- Society for Service to Urban Poverty, F-6/8A, Vasant Vihar, New Delhi, 110057, India

**Keywords:** IDUs, Depression, Suicidal ideation, HIV risk behaviors, India

## Abstract

**Background:**

Data on mental health among injecting drug users in South Asia is scarce yet poor mental health among users has significant implications for the success of HIV prevention and treatment programmes. A cohort of 449 injecting drug users in Delhi was examined on the following issues (1) examine trends in suicidal ideation, suicide plan and suicidal attempts over a 12-month period, (2) examine association between injecting practices (receive and give used syringes) and suicidal ideation over a 12 month study period.

**Methods:**

An observational study was conducted providing phased interventions with follow up interviews every 3 months to 449 injecting drug users (IDUs), from August 2004 to November 2005. The study was conducted in Yamuna Bazaar, a known hub of drug peddling in Delhi. Interventions included nutrition, basic medical services, needle exchange, health education, HIV voluntary counseling and testing, STI diagnosis and treatment, oral buprenorphine substitution, and detoxification, each introduced sequentially.

**Results:**

Suicidal ideation and suicide attempts, did not significantly change over 12 months of observation, while suicide plans actually increased over the time period. Keeping other factors constant, IDUs with suicidal ideation reported more giving and receiving of used syringes in the recent past. Conclusions**:** Mental health services are warranted within harm reduction programmes. Special attention must be paid to suicidal IDUs given their higher risk behaviours for acquiring HIV and other blood borne infections. IDU intervention programmes should assess and address suicide risk through brief screening and enhanced counseling.

## Introduction

Psychiatric morbidity among opiate users is well documented. Anxiety and depression are shown to be the most common psychiatric diagnoses among injecting drug users [1-3]. Suicidal behavior has also been found to be associated with opiate use [4,5], especially among injectors [6,7] and this along with overdose, are found to be the leading causes of premature mortality among young IDUs [8,9]. While it is difficult to establish the exact causal pathways, studies have consistently shown that depression [10-12] and lifetime admission to a mental health facility [13] are associated with suicidal behavior. Other factors found to be significantly correlated with suicidal attempts are sexual abuse [13], younger age, less education, poly drug use, recent heroin overdose [12], current suicidal ideation [12,14], less family support, and treatment seeking behavior [14-16].

In India, data on the prevalence of psychopathology among opiate users are scarce. Among the general Indian population the incidence of suicide was estimated to be 11.2 per 100,000 for the year 2011 (National Crime records Bureau, Ministry of Home Affairs). However, as is noted by Indian researchers, suicide rates estimated through police records are often under reported [17]. In fact, suicide rates have been demonstrated to be much higher than the national average in various studies ranging from 58 per 100,000 among young men [17] to 189 for 100,000 population above the age of 55 years [18]. Broader social and economic stresses are said to contribute to the majority of suicides in India, though mental illness does seem to be a risk factor in some proportion of suicides [19]. Indeed, family problems, illnesses, unemployment accounted for a large proportion of suicides in India in the year 2009 (National Crime records Bureau, Ministry of Home Affairs). Since suicide and depression are documented to be closely associated, it may be that these adverse conditions perhaps cause depression and lead to suicide. So far, the presence of suicidal ideation among drug users has not been a major focus of enquiry.

Historically, very few interventions in India targeting this group, whether drug treatment or HIV prevention, addressed mental health issues, despite evidence that depression among IDUs can compromise treatment outcomes or HIV prevention strategies. Depression can lead to a premature drop out and poorer prognosis after treatment [20] continued drug use [21] and heavier use [22,23] while in treatment. It has also been shown that depressed IDUs are more likely to engage in risky injecting practices [12,24-27]. These findings have implications for the success of HIV prevention programmes of a country. The present study, therefore, aims to: (1) examine trends of suicidal ideation and suicidal attempts across a 12 month period among a cohort of IDUs in Delhi (2) examine associations between recent injecting practices (receive and use syringes) and suicidal ideation over a 12 month period.

## Methods

An observational study was conducted in Delhi among 449 injecting drug users (IDUs) over a two year period from September 2004 to October 2006 in order to study the effectiveness of sequential interventions on a number of behavioral, biological and clinical outcomes among injecting drug users. The study was designed to provide phased interventions with follow up interviews every 3 months. Study participants were recruited for 15 months after which they were integrated into the ongoing intervention programme of Sharan which continued to offer oral buprenorhine substitution and referrals to general health and detoxification services. During the study period, participants were offered and underwent 6 follow up interviews.

### Data collection

**Figure 1:**
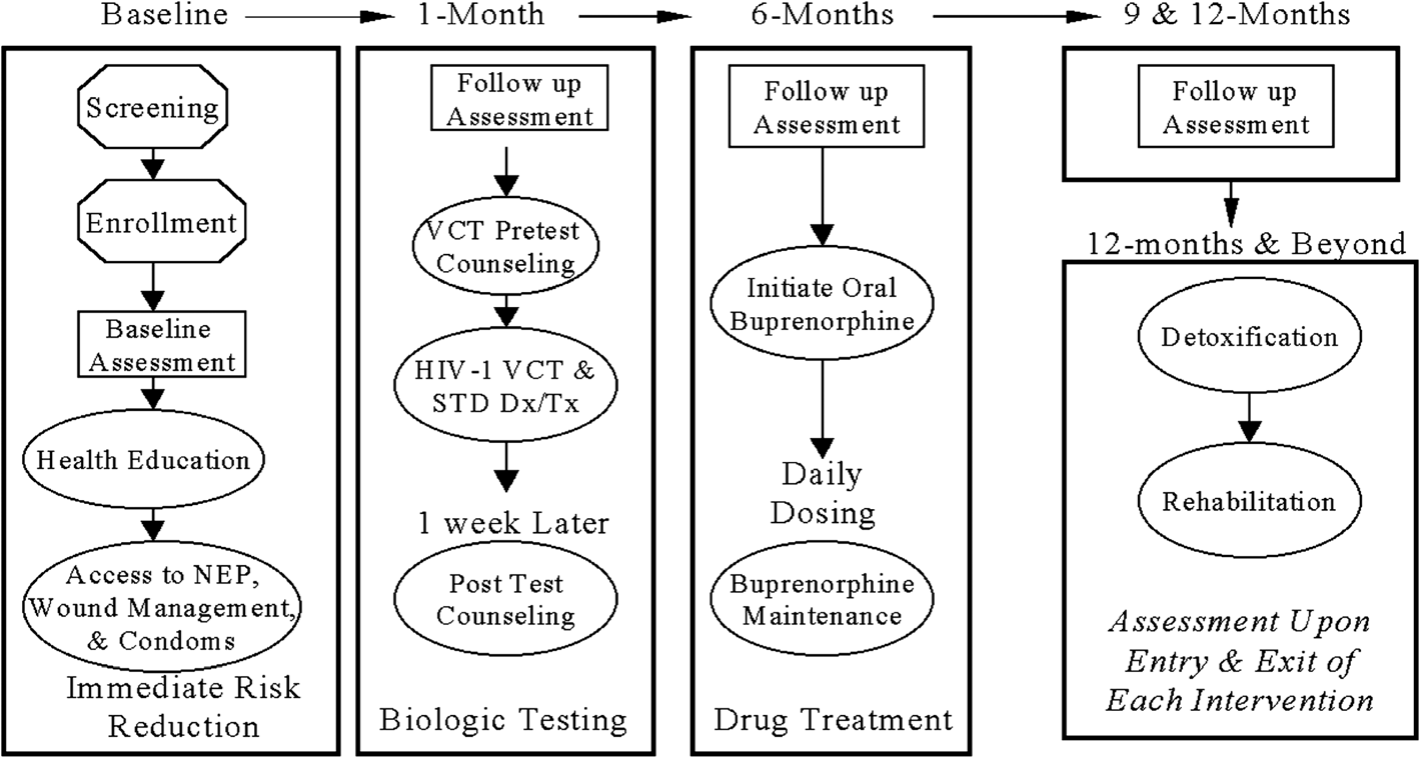
Phases of interventions and follow up interviews.

The study was conducted in Yamuna Bazaar, a known hub of drug peddling in New Delhi, India. The interventions as well as the interviews took place in a drop in centre, a free standing clinic established for the study. The baseline interviews were preceded by written informed consent. The study was approved by institutional review boards from both The Johns Hopkins University, Bloomberg School of Public Health, and The Society for Service to Urban Poverty (SHARAN). Each survey interview took from 45 minutes to an hour to complete, plus approximately 20 minutes for a brief health screening at each catchment. In accordance with the advice of the IRB, participants who expressed suicidal ideation, plan and attempt were provided with referral slips to the Institute of Human Behavior and Allied Science (IHBAS) in Delhi, a health care institute for promoting mental health. The questionnaires and medical forms did not include names but carried unique ID codes generated at the time of recruitment. Each participant was given a study ID card with their ID number and picture, and this was used to track utilization of services. Henceforth, the study participant was free to avail of the services that were offered at each stage of their participation. Figure 1 presents the phases of interventions provided to the participants:

Those successfully completing the detoxification program were eligible to participate in an inpatient rehabilitation program providing job training and continued social support. Ancillary services such as referral to shelter homes, bathing and shaving provisions were also offered.

Log books of interview appointments were maintained. Outreach workers attempted to trace participants who failed to turn up by visiting the places where they frequented the most (as most were homeless). Participants, who did not visit the drop in centre for a period of time either due to incarceration or ill health, were continued in the study if they reappeared within 4 months of the last interview. Those who disappeared for longer periods of time (more than 6 months) were allowed to reenroll from baseline, and we maintained a roster linking the ID numbers of these clients from each enrollment.

### Measures

The survey instrument assessed socio-demographics, drug use history, injecting and sexual risk behaviors, medical history, mental health status, family relationships, HIV knowledge, attitudes, risk perception, and experiences of discrimination and violence. Most measures were adapted from previous surveys used with the study population. The questionnaire was translated into Hindi, and then back-translated to English to check the accuracy of the translation. Both the translations were done by independent and separate translators. The Hindi questionnaire was then field tested among twenty IDUs. Questions on suicidal ideation, suicidal planning, and suicide attempts in the last 6 months were asked, in keeping with the definition of suicidal attempt, plan and ideation as posited by O’Caroll et al. [28]. To measure suicidal ideation, it was asked “During the last 6 months, have you ever had thoughts of taking your own life, even if you would not actually do it?” The question on suicidal plan asked “During the last 6 months, did you ever make a specific plan about how you would take your own life”, while that pertaining to suicide attempt asked “Have you ever attempted to take your own life in the last 6 months?” Only when the question on suicidal ideation was answered in the affirmative were the following questions on suicide plan and attempts asked.

### Data analysis

*Trends in suicidal ideation, suicide plan and suicide attempt:* Data from the baseline interview, the six-month interview, and the twelve-month interview were used to examine trends in suicidal behavior. In order to test the difference in proportions across follow up catchments, Cochran’s Q test was performed. The 15 month follow up was not considered for analysis as many study participants had graduated the study, or dropped out, and the sample would have been too small for meaningful analysis.

### Generalized estimating equations

For our second research question, we utilized generalized estimating equations (GEE) in order to assess the associations between suicidal ideation and recent sharing of used syringes (receiving and giving used syringes in the past 3 months). Because analyses of factors associated potentially with recent sharing included serial measures for each subject, we used GEE for binary outcomes with logit link for the analysis of correlated data. This approach allows for the identification of factors associated independently with the outcome during the entire study period [29]. In this analysis, we included all participants seen for baseline, six-month follow-up and twelve month follow-up interviews.

We considered the following independent variables associated with receiving and giving used syringes in the past three months: suicidal ideation (yes vs. no), formal schooling (yes vs. no), marital status (married/cohabiting vs. others), occupation (scavenging vs. others), income (up to Rupees 100 vs. more than Rupees 100) and age (continuous). All demographic variables referred to status at baseline interview and the suicidal ideation variable was in regard to three months prior to the interview. All p-values are two-sided. As a first step, we conducted univariate GEE analyses to determine factors associated with recent receipt and giving of used syringes. Next we entered all variables of interest into a fixed multivariate logistic GEE model. All statistical procedures were performed using SPSS software version 16.0 (SPSS, Chicago, IL, USA).

## Results

All 449 IDU participants were male. This is despite efforts made to attempt to recruit females, and it appears that there are few female IDUs in evidence. Most participants lived in and around the study area, and as indicated by previous ethnographic research, they had originally came to Delhi from nearby towns and villages as migrant workers [30]. Baseline data showed that the majority (89%) were homeless and lived on the street, earning an average daily income of Rs 107 (~$2), mostly from rag picking (91%). The median age was 28 years (IQR: 25–34 years). Less than half (35%) were married: close to 26% lived with their spouses and the rest were separated. The median age at first injection was 22 years (IQR: 19–29 years). All participants at baseline injected a combination of one or more of the following drugs: buprenorphine (91%); antihistamine (96%); diazepam (81%) and promethazine (21%). The mean injection frequency was 2.8 per day with 2 ml of each drug used at each injection episode. Slightly more than half (59%) reported using non injectable drugs along with injectables. Among the 240 participants (54%) who reported using drugs in groups, 66% said they shared drugs (n=160), 39% said they shared needles and syringes (n=95), and 65% shared drug equipment, such as mixing containers and ampoule bottles (n=158). IDUs who reported using drugs in groups also reported sharing food (86%) and sleeping space (74%). Almost equal numbers of IDUs admitted to ever giving and receiving a used syringe (41% said they had given a used syringe, 45% said they had received one). More than half (66%) had heard of HIV/AIDS, and knew of all the possible transmission routes. Sexual contacts in the last six months, mostly with sex workers, were reported by almost one third of the sample of IDUs. At the end of the study, there were 30 deaths (6.7% of cohort) reported among the entire cohort, the causes of death, except for road accidents, were unconfirmed. The prevalence of suicidal ideation, suicide plan, and suicidal attempts at baseline among this cohort are 23% (n=104), 18% (n=83), and 17% (n= 77) respectively. Only close to 3% (n=11) had ever been treated in a mental health institution.

*Trends in suicidal ideation, suicide plan and suicide attempt across study period*: The proportion of respondents reporting suicidal ideation and suicide attempt during the past six months did not significantly vary over the study period from baseline to 12 months (Table 1). A total of 366 respondents answered question on suicidal ideation at all three time points and about one-fourth of them responded affirmatively to this question. When asked about suicide plan, 6.0% (n=27) respondents answered at all three time points, out of which 74.1% (n=20) at baseline, 85.2% (n=23) at 6 months and 96.3% (n=26) at 12 months reported making a specific plan about taking their own life during the past 6 months. IDUs who reported attempted suicide at all three time points also stood at 6.0% (n=27), of which 70.4% (n=19) at baseline and 85.2% (n=23) at 6 months and 12 months reported having attempted to take their lives during the past six months of the survey. When comparing the groups across three time points for suicide plan we obtained a Cochran’s Q test of 6.7, df 2 with a P value of 0.034 (significant at 0.05 level). The difference in the proportions for suicidal ideation and suicide attempt were found to be non-significant.

**Table 1:** Proportion reporting suicidal ideation, suicide plan and suicide attempt at baseline, 6-month and 12-month interviews

	**Baseline**	**6-Month**	**12-Month**	**Analysis of proportions across follow up**^**a**^	**P-value**
Suicide Ideation (n=366)	89 (24.3%)	96 (26.2%)	93 (25.4%)	Q=0.497	0.780
Suicide Plan (n=27)	20 (74.1%)	23 (85.2%)	26 (96.3%)	Q=6.750	0.034*
Suicide attempt (n=27)	19 (70.4%)	23 (85.2%)	23 (85.2%)	Q=4.000	0.135

Note: N in each row represent the number of participants that responded at all three time points, thus included in the analysis of proportions.

^a^ tested with Cochran’s Q test, with 2 degrees of freedom.

*significant at 0.05 level.

*Factors associated with receiving and giving used syringes in last 3 months:* The univariate GEE analyses of sociodemographic and suicidal ideation associated with receiving used syringes are presented in Table 2. Factors found to be associated significantly in univariate analyses included suicidal ideation (yes versus no) [odds ratio (OR) = 1.11, 95% confidence interval (CI) 1.07–1.15]. In the multivariate GEE analysis, also shown in Table 2, factors that remained associated independently with receiving used syringes in our logistic model again included suicidal ideation (yes versus no) [adjusted odds ratio (AOR) = 1.09, 95% confidence interval (CI) 1.07–1.15]. In Table 3, the univariate and multivariate GEE analyses of variables associated with giving used syringes are presented. Suicidal ideation is found to independently associated with giving used syringes (OR: 1.12, CI: 1.08- 1.16). 

**Table 2:** Factors associated with receiving used syringe

	**Univariate GEE of factors associated with using of shared syringe**		**Multivariate logistic GEE of factors associated with using of shared syringe**	
**Characteristic**	**OR (95% CI)**	**P-value**^**a**^	**AOR (95% CI)**	**P-value**^**a**^
Suicidal Ideation	1.11 (1.07-1.15)	<0.001	1.11 (1.07-1.15)	<0.001**
yes versus no				
Formal Schooling	1.00 (0.96-1.04)	0.995	1.00 (0.96-1.05)	0.826
yes versus no				
Marital Status	1.02 (0.97-1.07)	0.511	1.01 (0.96-1.06)	0.626
married/cohabiting versus others				
Occupation	1.01 (0.97-1.06)	0.544	1.02 (0.98-1.07)	0.299
scavenging versus others				
Income (in INR)	1.03 (0.99-1.08)	0.139	1.04 (0.99-1.09)	0.086
100 versus more				
Age	1.00 (0.99-1.00)	0.747	1.00 (0.99-1.00)	0.867

Note: GEE: Generalized estimating equations, INR: Indian national Rupee, OR: Odds ratio, AOR: Adjusted odds ratio, CI: Confidence interval.

^a^ tested with wald chi square, 1 degree of freedom.

**significant at 0.01 level.

**Table 3:** Factors associated with giving used syringe

	**Univariate GEE of factors associated with sharing of used syringe**		**Multivariate logistic GEE of factors associated with sharing of used syringe**	
**Characteristic**	**OR (95% CI)**	**P-value**^**a**^	**AOR (95% CI)**	**P-value**^**a**^
Suicidal Ideation	1.12 (1.08-1.16)	<0.001	1.12 (1.08-1.16)	<0.001**
yes versus no				
Formal Schooling	0.99 (0.95-1.04)	0.755	0.99 (0.95-1.04)	0.767
yes versus no				
Marital Status	1.03 (0.98-1.09)	0.195	1.02 (0.97-1.08)	0.381
married/cohabiting versus others				
Occupation	0.99 (0.95-1.04)	0.810	1.00 (0.96-1.05)	0.795
scavenging versus others				
Income (in INR)	1.05 (1.00-1.10)	0.043	1.05 (1.00-1.10)	0.033
100 versus more				
Age	1.00 (0.99-1.00)	0.356	1.00 (0.99-1.00)	0.507

Note: GEE: Generalized estimating equations, INR: Indian national Rupee, OR: Odds ratio, AOR: Adjusted odds ratio, CI: Confidence interval.

^a^ tested with wald chi square with 1 degree of freedom.

**significant at 0.01 level.

## Discussion

A major finding of the study is the alarmingly high rate of suicide attempts and precursors to suicide among IDUs in New Delhi. As well, suicidal ideation and suicide attempts did not decrease over the study period; rather these measures remained constant throughout while the number of participants reporting suicide plan increased over the study period despite access to a broad range of health and IDU-risk reduction intervention services. These included nutrition, health education, oral buprenorphine substitution, regular health check ups, medical referrals, and detoxification and rehabilitation facilities. However, since we did not examine uptake of these services, we cannot definitively conclude that they did not improve the mental health of the suicidal participants. Also, the study period may not have been long enough to mark changes in mental health state of the participants. Despite these limitations, we can tentatively suggest that suicidal ideation did not decrease over a period that saw the introduction of several interventions including the provision of nutritious food which is generally found to be associated with high utilization. With regard to suicide plan, we did find a difference in proportion across the study period with an increasing number of respondents responding affirmatively to the question on suicide plan. Thus, what this suggests is that despite the services offered, more and more participants were reporting plans to commit suicide. Studies based in India demonstrate that unemployment, marginalization, financial difficulties, and the inability to buy food is associated with suicide [31-33]. Other studies have found homelessness to be a major factor in depression and other forms of psychopathology among IDUs [13,34]. Since most of our study participants are homeless, marginalized and suffer from an inability to buy food, using drugs in addition perhaps exacerbate the symptoms, thus necessitating mental health intervention through active referrals in harm reduction programs.

The fact that the majority of those with suicidal ideation reported attempting suicide, and that nineteen of these consistently reported attempts at all 3 time periods suggest, firstly, that suicidal ideation, in this population, can be a precursor of a suicide attempt [15] and secondly, that there is a small pool who consistently attempted suicide. Although a number of deaths occurred in the cohort during the study period (reported as adverse events to the IRB), we do not know whether they included suicides. However, the possibility cannot be ruled out, as there is no proper system of ascertaining the cause of death in India [19]. At least in one case, field workers verified that a study participant did commit suicide based on his earlier actions and statements which were witnessed by many.

We also saw that suicidal ideation and risk behaviors were associated consistently over the study period, albeit the modest effect sizes. Our findings find support with other studies that have shown a correlation between suicidal thoughts and risk behaviours, such as using syringes used by another [24], injecting more frequently [35], and sharing needles and syringes [12,25-27]. A harm reduction intervention has been operational for many years in Delhi, which is separate from the research study. The gains accrued in HIV prevention behaviours among IDUs subsequent to harm reduction interventions may very well be diluted if attention is not paid to this special section of the IDU population who present with suicidal behavior. In the context of comprehensive harm reduction programming, therefore, this would require an enhanced intervention targeting suicidal IDUs, as they continue with unsafe behaviours that make them and others vulnerable to infectious diseases.

Our study has a number of limitations. Since we could not determine if suicide is the cause of death among 8% of the cohort who died during the course of the study it leads us to propose that there is very real need for examining causes of mortality among this population, with the goal of prevention. We did not examine the uptake of services by suicidal IDUs, thus making it difficult for us to conclude that there is no improvement in mental health despite a broad array of services.

The findings tentatively suggest that HIV prevention efforts alone may not lead to reduction in behavioral risks among a certain group of IDUs. It may be necessary to provide services that ameliorate the conditions that lead to depressive symptoms and suicide which negatively influences HIV risk related behaviours. Linking harm reduction programmes with employment and income generating opportunities can help in mitigating economic hardships of IDUs, as well ensure stability in their lives. Furthermore, as drug use may also be contributing to their poor mental health, it is essential that the quality of detoxification and rehabilitation services be improved in order that IDUs may volunteer for such services. Additionally, it will be beneficial to have proper referral systems in place whereby IDUs exhibiting suicidal ideation and/or depression symptoms can access mental health services. Given the significance of the findings, it may be useful to develop simple mental health assessments that could be used in the context of harm reduction prgrammes to facilitate such referrals.

## Competing interests

The authors declare that they have no competing interests.

## Authors’ contributions

ES directed the operations of the study, drafted the manuscript and supervised the data analysis. BS managed the data and conducted the data analysis. LS supervised the data collection and gave inputs to the draft. MS advised the content and data analysis of the manuscript. All authors read approved the final manuscript.
